# Biophysical Determinants and Constraints on Sperm Swimming Velocity

**DOI:** 10.3390/cells11213360

**Published:** 2022-10-25

**Authors:** Carl D. Soulsbury, Stuart Humphries

**Affiliations:** School of Life and Environmental Sciences, Joseph Banks Laboratories, University of Lincoln, Green Lane, Lincoln LN6 7TS, UK

**Keywords:** spermatozoa, Reynolds number, biophysics, ejaculate traits, sperm length, sperm competition

## Abstract

Over the last 50 years, sperm competition has become increasingly recognised as a potent evolutionary force shaping male ejaculate traits. One such trait is sperm swimming speed, with faster sperm associated with increased fertilisation success in some species. Consequently, sperm are often thought to have evolved to be longer in order to facilitate faster movement. However, despite the intrinsic appeal of this argument, sperm operate in a different biophysical environment than we are used to, and instead increasing length may not necessarily be associated with higher velocity. Here, we test four predictive models (*Constant* *Power Density, Constant Speed, Constant Power Transfer, Constant Force*) of the relationship between sperm length and speed. We collated published data on sperm morphology and velocity from 141 animal species, tested for structural clustering of sperm morphology and then compared the model predictions across all morphologically similar sperm clusters. Within four of five morphological clusters of sperm, we did not find a significant positive relationship between total sperm length and velocity. Instead, in four morphological sperm clusters we found evidence for the *Constant Speed* model, which predicts that power output is determined by the flagellum and so is proportional to flagellum length. Our results show the relationship between sperm morphology (size, width) and swimming speed is complex and that traditional models do not capture the biophysical interactions involved. Future work therefore needs to incorporate not only a better understanding of how sperm operate in the microfluid environment, but also the importance of fertilising environment, i.e., internal and external fertilisers. The microenvironment in which sperm operate is of critical importance in shaping the relationship between sperm length and form and sperm swimming speed.

## 1. Introduction

Sperm competition occurs wherever ejaculates from two or more rival males compete to fertilise female ova [1,2]. This potent evolutionary force favours selection on traits that enhance male fertilizing ability. Over the last 50 years, many studies have shown that where sperm competition is greater, then traits such testes size are larger [3,4,5,6], ejaculate sizes and sperm concentrations are larger [7], ejaculate properties such as proportion of motile cells are greater [8,9,10] and sperm structural components such as midpiece length increase in size [6,11]. Taken together, these studies have shown sperm competition to be a key selective force acting on male reproductive traits.

Sperm swimming speed is one of the most important traits determining fertilization success [12,13]. Cross-species studies have found that where sperm competition is greatest, species have sperm that swim faster (e.g., fishes: [14]; birds: [15]; mammals: [16]). In turn, it is often assumed that selection for faster swimming speeds has driven an increase in length of sperm and many studies across [14,16] and within species [17,18] have shown a positive length-speed relationship. Nonetheless, within species the relationship between sperm length and velocity is not consistent (e.g., [19,20,21]) with several key factors thought to alter the relationship. Individual male quality [22] or reproductive tactics [23] can lead to changes in sperm speed without changes in morphology. Similarly, there may be trade-offs between shorter sperm swimming faster initially but exhibiting shorter survival [24]. Hence, we know that sperm swimming speed is an important plastic trait, but clearly do not yet understand what factors may shape this variation.

To consider what the expected patterns should be requires an understanding at a more mechanistic level. The long-held belief that total sperm length and velocity should correlate presents a biophysical puzzle, as this assumption is not expected from the physics governing objects at the microscale [25]. There is the general notion that the structural components of sperm scale linearly with size [16], but in fact the length of component parts such as midpiece length [26,27] and shape (head morphology: [28]), have been shown to be of specific importance and may scale and evolve independently [29]. Secondly, the component parts of sperm may trade-off against each other, for instance midpiece thickness and length [30], and it is clear that the fertilising environment (internal or external) will be important in altering these relationships [18,31,32]. Thirdly, patterns are not always supported theoretically or empirically. For example, larger heads may increase drag [25], and several within-species studies have shown that shorter sperm may in fact swim faster [24,33]. Hence, the generality of relationships between sperm velocity and morphology are still unclear.

Sperm operate in a different physical environment to that which we are used to [25]. Firstly, cells and organisms (e.g., bacteria, algae) that swim at microscales (<10 µm) are governed by the hydrodynamics of the embedding fluid and the surface boundary of surrounding structures (female reproductive tracts, ova surface). At this scale, inertia is unimportant and the Reynolds number, a dimensionless ratio of inertial forces to viscous forces, is low [25,34]. Movement in this regime can be likened to a human swimming in molasses. Moreover, sperm swim via pushing so that the power generation component (flagellum) is located at the rear and the passive load (head/nucleus) is at the front [35]. Component sperm structures such as head shape, can also be important with, for example, many passerine sperm possessing a structural modification to the midpiece in the form of a mitochondrial helix that extends from the head and spirals distally down the flagellum ([36]; but see notable exceptions: [37]). This leads to a “twist-drill” swimming motion [38], which contrasts with the basal sliding pattern found in mammals [39], but see [40].

Despite theoretical and empirical efforts, how sperm competition affects sperm swimming speed remains controversial [19,41]. One key future aim would be to demonstrate general principles governing sperm length-swimming speed relationships that allow testable predictions across and within species [42]. In this paper, we set out a biophysical framework that provides a set of testable mechanistic predictions on how sperm length and velocity might be interrelated. We then use data from across 141 species to show empirical evidence that the power output that is scaled to sperm length places a limitation on sperm velocity in internally fertilising sperm.

## 2. Materials and Methods

### 2.1. Empirical Data

We collected empirical data from the published literature on sperm velocity (VCL, curvilinear velocity; VSL, straight-line velocity; VAP, average path velocity). We chose single species values from single studies, rather than pooling means from multiple studies to avoid between-study variation in measurement conditions. Factors such frame rate in CASA (computer-assisted semen analysis [43]), the media used [44], as well as inter-specific variation from population and genetic differences [45,46] all influence sperm velocity independently of sperm morphology. Such differences contribute to undoubted variation within our dataset; by keeping single species values, we reduce the potential unexplained variation from within-species, though may increase some between-species variation. This may reduce our analysis power and increase noise within our dataset. We preferentially selected studies where sperm measurements were given or could be measured from (see below), those studies with velocity measurements from fresh samples and where we could find measurements VCL, VSL and VAP. For some species there are multiple available studies with velocity measures and in this case, a study was chosen at random so as to avoid bias in selecting velocities that matched our hypothesis. Our priority was to increase the number of species. In contrast to sperm velocity, measurements of sperm size (especially width) were a major limiting factor (see below).

For our analysis we used VCL only, as we had largest sample size for this measure, and felt this could remove factors affecting sperm trajectory. In any case, measures were significantly correlated (Spearmans rank correlations VCL & VSL: *r*_s_ = 0.61, *p* < 0.001, VCL & VAP: *r*_s_ = 0.68, *p* < 0.001; VCL & VSL: *r*_s_ = 0.61, *p* < 0.001, VSL & VAP: *r*_s_ = 0.81, *p* < 0.001). We extracted data on sperm morphology (total length (µm), head length and width (µm), midpiece length and width (µm), flagella length (µm)) from published values, or where necessary from SEM micrographs and light microscope images both from published and unpublished sources. Some terminology to described sperm structure is used variably across the literature. Here, we define head length as including the acrosome and nucleus. The midpiece is the main power generation unit for the cell and midpiece length is the area containing the mitochondria that power cell movement. In mammals, this area is terminated at the annulus but some taxa lack the annulus and the mitochondria can extend helically down the axoneme, e.g., passerines [47]. The flagella forms the main power output for the cell, and includes the principal and end pieces. In many passerines for example, the flagella is partly covered by the midpiece. Wherever possible, we used sperm morphology data and velocity data from a single source, but in numerous cases, data had to be combined from separate studies. There will also be cases where measurements from the literature refer to structures that are unclear; clear adherence to a consistent terminology, e.g., [47] is vital. Data are shared on FigShare (doi:10.6084/m9.figshare.15170256).

### 2.2. Biophysical Models

Sperm are generally small and slow enough that they operate in a low Reynolds number regime [25] where relative speed (*u*) is directly proportional to the force (*F*) generated by the propulsive mechanism (in this case the flagellum). In this regime Stokes law can be used, meaning that for a spherical object F=6πηru where *η* is the viscosity of the surrounding fluid, and *r* is the radius of the sphere. This relationship can be rearranged to show that speed is equal to the ratio of force to viscous drag (u=F/6πηr). Generalizing for a non-spherical shape, we can say that u∝F/ηL, where *L* is the total length of the cell and remembering that in the case of a spermatozoan, the viscous drag (in this case frictional resistance, ηL) will depend on the shape and size of the whole sperm, as well as hydrodynamic interactions between its parts [42,48]. However, to simplify we assume that the viscous drag is proportional to our length measure *L* (see Supplementary Materials for a justification of this assumption on both scaling and data grounds). Throughout we use the notation *X_y_* to denote a dimension (*X*) of a specific morphological component (*y*), for instance flagellum length (*L_f_*) or midpiece volume (*V_m_*).

Given that the power required to move an object is equivalent to the product of speed and force, the power required to move our object through a fluid medium can be approximated by P=Fu. If we further decompose the force *F* into the frictional resistance to movement of the object multiplied by velocity (remembering that F∝ηLu) we obtain $P\propto \eta Lu^{2}$. Following from our generalisation of Stokes law, and treating viscosity as constant (allowing us to ignore its effect in this model), we see that the swimming speed of our spermatozoan is proportional to the square root of the ratio of power to our length measure so that,

(1) $$u\propto \sqrt{\frac{P}{L}}$$

**Figure 1:**
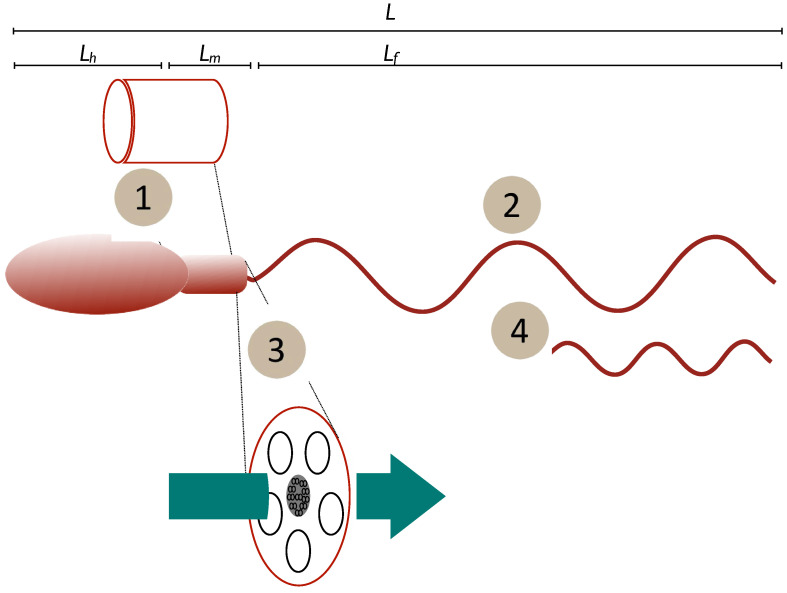
Schematic of potential relationships tween form and function. Mechanisms may be (1) mitochondrial/midpiece volume as a proxy for energy storage, (2) length of the power generator (flagellum), (3) midpiece or flagellum cross-section as a proxy for rate of diffusive transport of ATP and the creatine phosphate (CrP) shuttle or (4) power output that varies due to flagellar or dynein dynamics to maintain a constant force. Length measures: *L*—total cell length; *L_h_*—head length, *L_m_*, midpiece length, *L_f_* —flagellum length.

Using this simple framework, we can look at a number of hypotheses and theories relating to the relationships between functional morphology and performance. Several non-exclusive hypotheses exist (e.g., [25,42,48,49,50], but we can also draw on the work of Dusenbery [51,52] whose work on gametes in general is illuminating. As a starting point, we consider four scenarios (Figure 1):

(1) *Constant power density*. Power output is determined by body size alone and the power output of the spermatozoan is determined by its midpiece volume (*V_m_*) multiplied by a constant power density (Power per unit volume = constant). Dusenbery [52] discusses the influence of chromatin volume on this value, but the current work is a broad-brush approach. In this case, we can say that energy generation by the midpiece is the limiting factor determining speed.
(2) *Constant speed*. Power output is determined by the flagellum and so is proportional to flagellum length (*L_f_* a component of total length *L*). Here, the rate of transfer of energy to the surrounding fluid is limited by the length of the flagellum.
(3) *Constant power transfer*. Power output is determined by the rate of energy transfer from the midpiece to (and along) the flagellum. As diffusion is involved this should be dependent on the cross-sectional area of the flagellum ($r_{f}^{2}$) and/or midpiece ($r_{m}^{2}$). For this case the rate at which energy is transferred to the flagellum is the limiting factor (see Supplementary Materials for further discussion).
(4) *Constant force*. Power output is variable, but the propulsive mechanism is optimised to generate the same force, independently of the size of the spermatozoan. Derived [52] from experimental observations of sperm and flagellated microorganisms where increasing viscosity of the surrounding fluid leads to slower swimming speeds (e.g., [53]), implying that only a constant force is available.

If we assume that the volume of the sperm (*V*) or its midpiece (*V_m_*) is proportional to the cube of its length ($V\propto L^{3}$ and $V_{m}\propto L_{m}^{3}$), that flagellum length is linearly and directly proportional to total length ($L_{f}\propto L$), and that cross-sectional area of any sperm component is proportional to the square of its length ($A_{y}\propto L_{y}^{2}$) we can formalise the four scenarios as follows:

*Scenario (1): Constant Power Density*

For constant power density we assume that power output is directly proportional to the energy stored in the sperm of its component parts and that this is in turn proportional to the volume of the sperm or component ($P\propto V_{m}\propto L_{m}^{3}$, Figure 1. #1). In this case, we substitute this relationship into Equation (1) to see that,

(2) $$u\propto \sqrt{\frac{V_{m}}{L}}\propto \sqrt{\frac{L_{m}}{L}}\propto \frac{L_{m}^{3/2}}{L}$$

so that speed is proportional to a ratio between the square root of midpiece volume and total length.

*Scenario (2): Constant speed (power proportional to flagellum length)*

For power proportional to flagellar length (Figure 1. #2), either flagellar length is directly proportional to total length, and so

(3) $$u\propto \sqrt{\frac{L_{f}}{L}}\propto \sqrt{\frac{L}{L}}\propto 1$$

or we can add a further specification that flagellar length is always less than the sperm total length, in which case

(4) $$u\propto \sqrt{\frac{L_{f}}{L}}\underset{L_{f}<L}{\lim}<1$$

In both cases speed is constant (independent of *L*), giving a mechanistic explanation for Dusenbery’s constant speed model.

*Scenario (3): Constant power transfer*

For power proportional to cross-sectional area (Figure 1 #3) we use the square of the flagellar radius ($r_{f}^{2}$) and find,

(5) $$u\propto \sqrt{\frac{r_{f}^{2}}{L}}\propto \frac{r_{f}}{\sqrt{L}}$$

so that speed is proportional to a ratio of the flagellar radius to the square root of length. We use flagellar cross-sectional area here but note that this is likely to be proportional in some sense to a generalised sperm cross-sectional area. In our data set we find a correlation between midpiece radius (a proxy for flagellar radius) and head radius (PGLS: F_1,139_ = 15.71, *p* = 0.0001).

*Scenario (4): Constant force*

For constant force (from u∝F/ηL, Figure 1 #4) we obtain

(6) $$u\propto \frac{F}{L}\propto \frac{1}{L}$$

so that speed is (perhaps surprisingly) proportional to the inverse of sperm length.

## 3. Statistical Analysis

Sperm structure, and particular the scaling of individuals parts is variable across the animal kingdom [54]. To avoid this differing base structure confounding models, we identified clusters of morphologically similar sperm based structural measurements (total sperm length, head length, head width, midpiece width, midpiece length, flagella length) using a combination of principal components analysis (PCA) and hierarchical cluster on principle components (HCPC) analysis using the FactoMineR package in R 4.0.3 [55,56]. We examined scree plots to determine which principal components were retained for cluster analysis.

**Figure 2:**
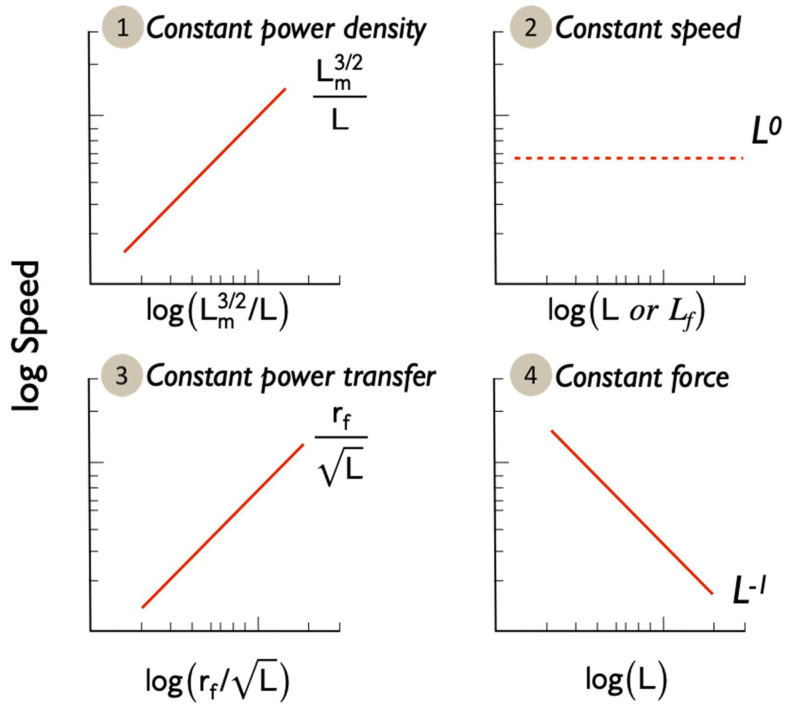
Predicted relationships between swimming speed and morphology for the four hypotheses. Note the Log-Log axes.

Based on hierarchical cluster analysis, we identified five distinct clusters. Within each cluster, we fitted a phylogenetic generalized least square regression (PGLS) models implemented in the R 4.0.3 [56], using the packages *ape* [57] and *mvtnorm* [58]. We used a time calibrated tree from TimeTree (http://www.timetree.org) but were not able to include nine species (*Arenicola marina, Galeolaria caespitosa, Anthocidaris crassispina, Catla catla, Labeo calbasu, Lota lota, Tilapia zillii, Tegillarca granosa, Ficopomatus enigmaticus*) as they could not be placed in the tree, even by swapping in sister clades. We calculated the phylogenetic scaling parameter Pagel’s lambda (*λ*), which measures statistical dependence due to phylogenetic relationships, differed from 0 [59]. We first tested the relationship between VCL and total sperm length for each sperm cluster for comparison to many other studies. We then tested the fit of predicted slopes from Equations (7)–(10) that relate to Equations (2)–(6), and which are illustrated in Figure 2. We determined a fit of the regression slope to the model prediction if the 95% CI of the log-log slope overlapped the predicted slope. Log-log relationships enable expression of proportionality rather than direct linear relationships and the regression models for each hypothesis were:

(7) $$\mathrm{Constant}\;\mathrm{power}\;\mathrm{density}\;VCL\times L\propto L_{m}^{3/2}$$

(8) Constant speed VCL∝Lf

(9) $$\mathrm{Constant}\;\mathrm{power}\;\mathrm{transfer}\;VCL\times \sqrt{L}\propto r$$

(10) $$\mathrm{Constant}\;\mathrm{force}\;VCL\propto \frac{1}{L}$$

## 4. Results

### 4.1. Sperm Structural Variation

PC1 accounted for 60.85% of the variation and predominantly captures variation in sperm length (Table 1). PC2 and PC3 accounted for 16.79% and 13.36% of the variation, respectively, and predominantly captured variation in head (PC2) and midpiece (PC3) width (Table 1).

**Figure 3:**
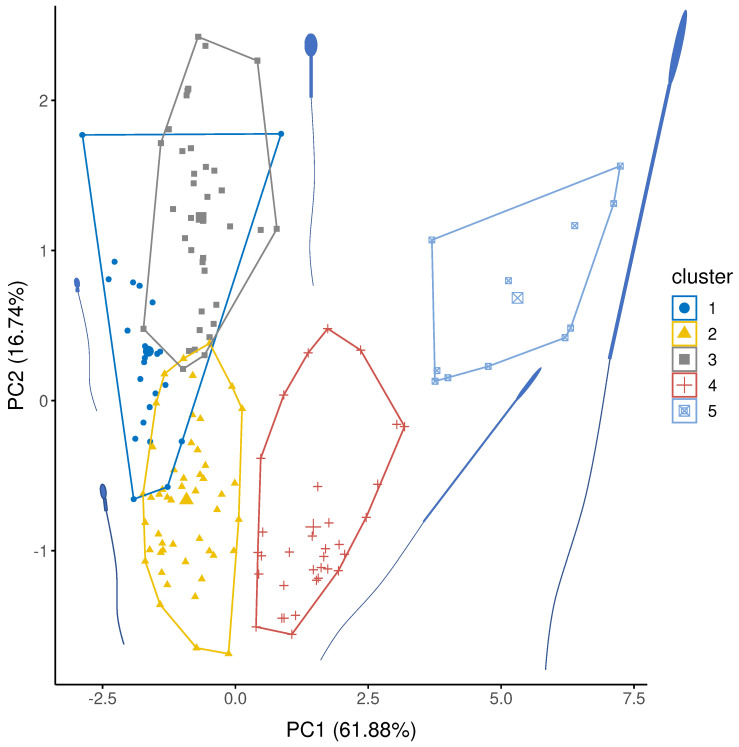
Cluster analysis showing the five representative groupings of sperm structure for PC1 and PC2. PC1 explains variation in sperm component lengths (explained variation = 61.88%) and PC2 explains variation in sperm head width (explained variation = 16.74%: Table 1). Average sperm morphology for each group are presented. Sperm sizes and component are scaled to each other and represent the mean size for each cluster.

Sperm were grouped in five distinct clusters (Figure 3) using hierarchical clustering of factor scores from the three retained principal components (Table 1). Cluster 1 were characterised by relatively short sperm with short, fat midpieces; 23 species were classified as cluster 1 of which 21 were external fertilisers: 13 species were fish, 8 species were molluscs or marine worms. Cluster 2 was similar to Cluster 1, but with slightly longer sperm with longer but thinner midpieces. Cluster 2 contained 49 species, including 29 species of fish and 12 mammals. Cluster 3 were characterised by short heads relative to midpiece length; of 37 species in Cluster 3, 36 species were mammals. Cluster 4 was characterised by long headed thin sperm, with a long midpiece relative to the flagellum. Most species in cluster 4 are birds (27/33 species) and so, here the midpiece is encasing a large proportion of the flagella. Three species of reptile and three species of rodent also have cluster 4-type sperm. Finally, Cluster 5 sperm were extremely long and thin with long midpieces relative to the flagellum. Here, most species were birds (10/11) with one rodent species.

### 4.2. Sperm Structure and Velocity

Within sperm clusters, only Cluster 2 (PGLM: F_1,42_ = 5.62, *p* = 0.022) showed a significant relationship between total sperm length and VCL. For all other clusters, there was no significant relationship (Cluster 1: PGLM: F_1,15_ = 0.78, *p* = 0.392; Cluster 3: PGLM: F_1,34_ = 0.07, *p* = 0.788; Cluster 4: PGLM: F_1,30_ = 0.16, *p* = 0.690; Cluster 5: PGLM: F_1,9_ = 0.09, *p* = 0.773).

**Figure 4:**
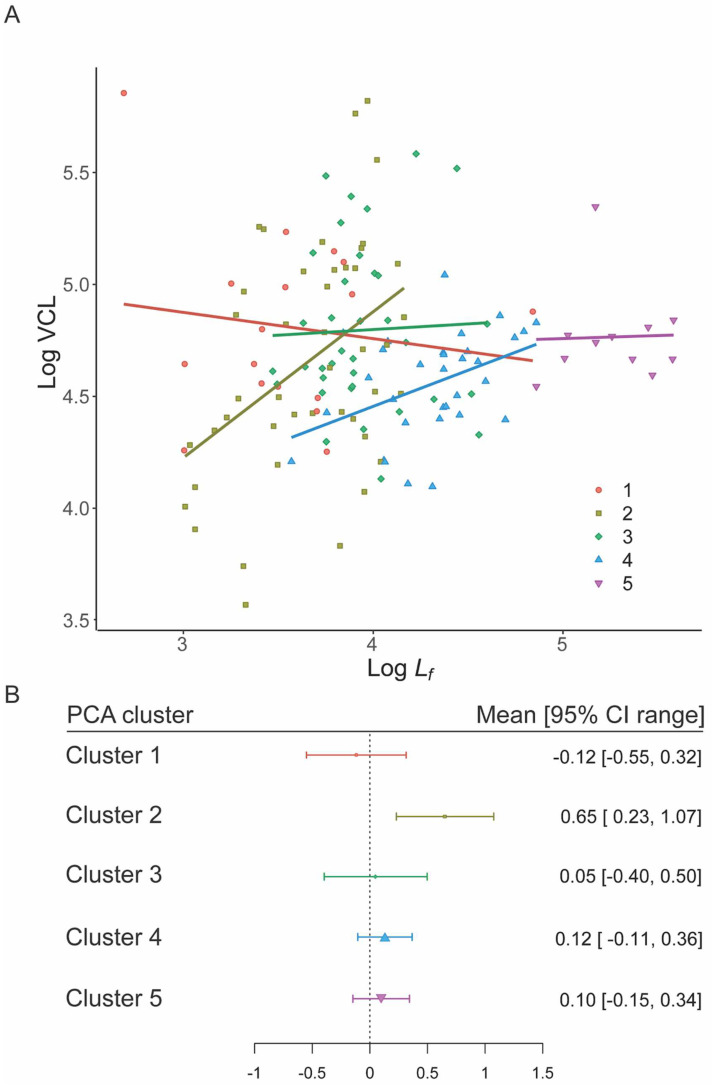
Speed–Flagellum length relationships for the five PCA morphology clusters. (**A**) PGLM slopes for each cluster. (**B**) Distribution of mean and 95% CIs for cluster slopes, point size is proportional to *n*. Only the slope for cluster 2 differs from the expected value of zero (Constant speed model) based on 95% CI overlap.

There was a clear overlap with predicted slopes for hypothesis 2 (constant speed) in Clusters 1, 3, 4 and 5 (Table 2), whilst Cluster 2 did not overlap zero. From this we infer evidence for selection on energy transfer in Clusters 1, 3–5 (Figure 4A,B) as in the Constant speed model the rate of transfer of energy is limited by the length of the flagellum. There was no support for hypotheses 1, 3 or 4 amongst any of the Clusters.

Since Cluster 2 was unusual in containing both external and internal fertilizing species, we re-ran hypothesis 2 (constant speed) for each grouping; we found that internal fertilisers overlapped zero, supporting the constant speed model, while external fertilisers did not (Figure S4).

## 5. Discussion

Sperm ultrastructure is known to show incredible diversity, especially in the size or even absence of component parts [54]. In our sample, we identified five clusters that varied clearly across the dimensions of sperm components, and importantly across the width. Specifically, Cluster 1 (mainly fish), Cluster 2 (a mix of fish and mammals) and Cluster 3 (mammals) conform to a typical ‘head plus midpiece’ arrangement (Figure 1), but mainly differ from each other in that they are internal (Cluster 3) and external (Cluster 1) fertilisers. Fertilisation mode may be expected to create differences in the relationship between sperm size and velocity [18]. Interestingly Clusters 4 and 5 mostly contained longer avian sperm (mainly passerines) and a handful of rodents, but all are internal fertilisers. This general separation of long and short sperm (as well as thin versus thick sperm) in our PCA clusters supports the idea that the internal fertilising environment selects for longer sperm [32,60].

In our analysis of we found two key outcomes. We did not find a positive relationship between total sperm length and VCL, again emphasizing the general the mix of studies where total sperm length and sperm velocity do [16,61] or do not relate to each other [19,20,21]. Sperm length in each of our clusters was driven almost entirely by flagellum length (Supplementary Figure S1), which could suggest that this is a key component of sperm speed. However, when we consider model predictions for limitation via scaling of power output (where power output, but not speed, depends on flagellum length: scenario 2, eqns. 3 and 4) we see no clear relationship between VCL and flagellum length in all clusters except for Cluster 2 (Table 2). This suggests that the limiting mechanism across these four clusters is the output of power through the flagellum to the surrounding fluid, supporting Dusenbery’s [52] constant speed model (our scenario 2), an intermediate between a constant force (our scenario 4) and a constant power density model (our scenario 1). Dusenbery [52] proposed this as an intermediate between two mechanistic models, but here we have illustrated a potential mechanism to raise this above a phenomenological model. In this case, and irrespective of fertilization mode, power output is determined by the number of motor units making up the flagellum itself as well as the drag of the flagellum, and both are directly related to the length of the flagellum. Crucially, however, this constant speed model also incorporates the relative relationship between flagellum and head length (here as *L_f_/L*) as suggested by Humphries et al. [25].

That Clusters 3–5 are characterised by internal fertilisation suggests to us that flagellum length might be a key selective pressure because of the direct interaction between the internal walls of the female reproductive tract and the likelihood [62,63] that the fluids in these tracts are viscoelastic, rather than Newtonian as assumed in our modelling. Newtonian fluids respond linearly when a stress is applied, while elastic materials return to their original state once a stress is removed. Viscoelastic materials such as mucus react in both these ways and so exhibit time-dependent stress responses. Our models may well not capture additional considerations such as hydrodynamic interactions between sperm components or specific morphological differences that could explain the positive but non-linear relationships seen between VCL and $L_{M}^{3/2}/L$.

Our models assume a relative constant viscosity whereas the fertilising environment, both internal and externally, can vary massively [64] and is vital in shaping key movement traits such as flagellar waveform [65]. We know that in mammals and birds, the velocity of sperm is decreased in more viscous environments [44,64,66], and “twist-drill” motions of bird sperm seem to have a smaller decrease in velocity, at similar levels of viscosity [44,66]. Additionally, sperm swimming speed is only one component of velocity, which can include swimming trajectory, something that is also impacted by fertilization environmental and the viscosity of the surrounding media [67]. This is also suggested by theoretical work examining size and shape of a spermatozoon and the hydrodynamic interactions between its parts [42,48], especially as this may lead to fundamental differences in performance in situ (e.g., [68]). Furthermore, our models consider a simple head-midpiece-flagella form of sperm, whereas sperm shape is incredibly diverse. For most of our data, sperm construction is similar, but gross changes to sperm morphology, e.g., head shape in passerines or presence of fins on sturgeon sperm can change the hydrodynamic properties of the sperm [69].

In a similar way, our assumption of constant speed allows us to ignore the effect of beat frequency (and to a second order effect beat waveform), simplifying the analysis and restricting it to morphological parameters. While beat frequency can clearly be important [70], there is currently not enough data to include frequency in any useful analysis. From the limited data available it does seem that frequency is relatively constant unless sperm are hyper-capacitated or experience viscosity changes (e.g., [53]). Studies of the former usually ignore the pre-fast swimming phase in data collection, and we have avoided using data from studies involving viscosity.

Taken together, our results reemphasise the critical role of the fertilizing environment in shaping sperm morphology [54] and in turn the complex relationship between sperm morphology and sperm velocity. We suggest that viscosity is a fundamental driver in changing sperm structure and in turn plays a vital role in shaping the biomechanical movement of the sperm. Future work should therefore focus more specifically on sperm velocity and the interactions with the fertilising environment. Additionally, much of the data used in this paper comes from vertebrates (mainly birds, fish, mammals). It is imperative that we expand our data collection to a greater range of taxa, so we can clearly understand the interactive forces between sperm size and shape, the fertilizing environment and the outcomes for sperm movement.

For those seeking to understand the morphology-velocity relationship for a given system these results complement those of Humphries et al. [25]. The more detailed set of analyses presented here begin to address the mechanisms that may drive selection for differing sperm morphologies based on energetics of sperm movement. Humphries et al. [25] aimed to help explain the variation seen in sperm morphology-speed relationships by highlighting the relative importance of the head and flagellum and any iso- or allometric relationship between them. Here, we take this thinking forwards by examining some of the patterns underlying the simple shape-drag relationships examined in Humphries et al. to provide a number of testable hypotheses. We note that the explicit links to the Humphries et al. model are that with our assumption of linear scaling of drag and length the constant speed model incorporates the head to flagella ratio examined in Humphries et al., it is also true that head length variation adds to drag and this is to some extent captured in our PCA.

Our results suggest that for cross-species studies similar patterns may underly the variation seen in length-velocity relationships as for within-species analyses. With this in mind, work is still needed to explore the implications for variation in the scaling between sperm parts (we assume here mostly linear relationships). Our suggestion would be that all four models are tested with within-species data, but that there is perhaps more emphasis on the Constant Speed (as supported by our results) and Constant Force models (where our results hint at a link with Cluster 2).

## Figures and Tables

**Table 1:** Individual weightings for sperm size measurements, the individual % variance, cumulative % explained variance and eigen values of the three top principal components.

Parameter	PC1	PC2	PC3
Total sperm length	−0.490	0.237	−0.087
Head length	−0.444	−0.025	0.122
Head width	0.154	0.826	0.529
Midpiece length	−0.485	0.081	−0.143
Midpiece width	0.252	0.462	−0.812
Flagella length	−0.491	0.202	−0.133
% variance	61.88	16.74	13.40
Cumulative variance (%)	61.88	78.62	92.02
Eigen value	3.71	1.00	0.80

**Table 2:** PGLM slopes for each morphology cluster. Slopes in **bold** indicate overlap of 95% CI with predicted slope. λ estimates phylogenetic signal, with λ = 0 inferring no phylogenetic signal in the data and λ = 1 corresponding to a Brownian Motion model of evolution.

Model	Prediction	Cluster 1 Slope [95% CI] *n* = 17	λ	Cluster 2 Slope [95% CI] *n* = 44	λ	Cluster 3 Slope [95% CI] *n* = 36	λ	Cluster 4 Slope [95% CI] *n* = 32	λ	Cluster 5 Slope [95% CI] *n* = 11	λ
1. Constant power density	β = 1	−0.02 [−0.18, 0.13]	0.00	−0.02 [−0.12, 0.09]	0.00	0.13 [−0.09, 0.35]	0.00	0.00 [−0.09, 0.09]	1.00	0.03 [−0.18, −0.25]	0.00
2. Constant speed	β =0	**−0.12** **[−0.55, 0.32]**	**0.00**	0.65 [0.23, 1.07]	0.00	**0.05** **[−0.40, 0.50]**	0.00	**0.12** **[−0.11, 0.36]**	0.00	**0.10** **[−0.15, 0.34]**	1.00
3. Constant power transfer	β =1	0.24 [−0.33, 0.81]	0.00	0.02 [−0.45, 0.48]	0.00	−0.05 [−0.43, 0.32]	0.00	−0.13 [−0.15, 0.40]	0.00	−0.08 [−0.28, 0.12]	1.00
4. Constant force	β = −1	−0.17 [−0.56, 0.21]	0.00	0.50 [0.09, 0.91]	0.00	−0.07 [−0.60, 0.45]	0.00	0.06 [−0.21, 0.33]	1.00	0.04 [−0.25, 0.33]	1.00

## Data Availability

Data are available on FigShare (doi:10.6084/m9.figshare.15170256).

## Supplementary Materials

The following supporting information can be downloaded at: https://www.mdpi.com/article/10.3390/cells11213360/s1, Figure S1: The relationship between proportion of total sperm length taken up by the flagella in relation to the 5 sperm clusters; Figure S2: The relationship between total (*L*) and flagellar (*L_f_*) lengths in our dataset; Figure S3: Simulation results for the relationship between ‘drag component’ and total cell length; Figure S4: Speed – Flagellum length relationships for the internal and external fertilizers in PCA morphology cluster 2. Supplementary File: Simplifying assumptions [25,30,48,51,52,71,72,73,74].
