# Supplementary material for: Biophysical Determinants and Constraints on Sperm Swimming Velocity
Source: Cells. 2022 Oct 25;11(21):3360. doi: 10.3390/cells11213360 (PMC9656961; doi:10.3390/cells11213360)
Supplement: Supplementary file 1 [file cells-11-03360-s001.zip › cells-1341539-supplementary.pdf]

Supplementary material

Biophysical Determinants and Constraints on Sperm Swimming Velocity

Carl D. Soulsbury * and Stuart Humphries

School of Life and Environmental Sciences, Joseph Banks Laboratories, University of Lincoln, Green Lane, Lincoln LN6 7TS, UK
* Correspondence: csoulsbury@lincoln.ac.uk

Simplifying assumptions

Source of ATP for swimming

Our model assumes that all ATP for swimming is generated in the midpiece. We therefore consider the cross-sectional area of the midpiece as a limiting factor as this is the area through which any ATP or shuttles must pass in order to deliver energy to the flagellum. This cross-sectional area represents a bottleneck as diffusion of material will be along the midpiece and into the flagellum (see also the legend for Figure 1). Diffusion and kinase shuttles such as Creatine Phosphate or adenylate kinase have been shown to be enough to supply length of flagellum [1] and we argue that the length of the flagellum can be ignored in this instance. It is difficult to see how the rate of delivery of ATP would vary with flagellum length unless the density of shuttle molecules or their speed declines along the length of the flagellum.

The assumption that the midpiece is the source of all ATP for swimming may be a contentious issue and [1] also argue that multiple strands of evidence demonstrate that alternative mechanisms for ATP delivery to the flagellum must exist. We agree that the source of ATP may vary between species, but even if substantial ATP is generated via glycolysis within the flagellum, then it seems likely that the cross-sectional area of the flagellum may be important in those species. However, by further assuming (as with diffusion of ATP) that glycolytic activity does not vary along the length of the flagellum we can again ignore flagellum length for this particular analysis.

In a similar vein, in Scenario 3 (Constant power transfer) our focus on flagellum width (a proxy for its cross-sectional area) is based around the idea that diffusion is often considered in term of flux. Thus we consider the movement of material per unit area, and it for this reason that we concentrate less on flagellar length than cross-sectional area. In this instance we are assuming that in general diffusion and shuttles are adequate to deliver ATP but *only* if enough ATP is delivered from the mitochondria to the flagellum, which will in large part be determined by the cross-sectional area of the interface between the two.

Drag proportional to length

Our simplification is based ultimately on the assumption that sperm can be approximated as a rigid ellipsoid (similar to that made for bacteria in

[2,3]). Although sperm rarely have truly ellipsoid forms this geometry can approximate most shapes (think of a needle through to a ball). Dusenbery also points out that "…chemists use the ellipsoidal model to study the shapes of molecules … although molecules are even less like ellipsoids than are bacteria" [3] (p5981).

More formally, it can be shown [4] that for a prolate ellipsoid extended to a needle-shaped rod (longest axis >> shorter axis, i.e., $L_1 >> L_2$) the force acting in a direction parallel to its axis L_1 at velocity u is given by

$$F = \frac{4\pi\eta a u}{ln(L_1/L_2)+0.193} \qquad \text{S1}$$

while a generalisation for both oblate and prolate ellipsoids is

$$F = 6\pi\eta u L K\left(\frac{L_2}{L_1}\right) \qquad \text{S2}$$

Where $L = L_2$ for an oblate ellipsoid and $L = L_1$ for a prolate ellipsoid. The numerical values and a correction factor K are given for different ratios L_2/L_1 in [5], but in terms of proportionality we can say that

$$F \propto \eta L_1 u \qquad \text{S3}$$

as used in our model.

Another approach is to model the drag of the head and flagellum seperately (e.g. *[6]*) such that

$$F = (6\pi\eta r + K_T L_f)u \qquad \textit{S4}$$

where

$$K_T = \frac{2\pi\eta}{log(2L_f/a)} \qquad \textit{S5}$$

For a flagellum of length L_f and radius a. However, while this method should provide a more accurate estimate of drag, as the relationship between L_h and L_f is unclear both within and between species, exact solutions to the two drag components are required. This approach therefore severely restricts the size of the available dataset and, due to the the two separate drag terms, does not lend itself to generalisation and scaling arguments. For our purposes the increased accuracy of estimating the drag for both the head and flagellum separately is offset by the complexity of constructing scaling arguments from additive components. It also introduces head and tail morphology-specific parameters that are both hard to deal with analytically and require morphology measurements with which to test the models that are not easily available.

Scaling of sperm component morphology

As highlighted above, a consistent issue when studying cross-species morphology-performance relationships in sperm is limited data on sperm component dimensions and, as a result, limited understanding of the scaling of different components to each other [7].

For the purposes of our modelling, we assume a linear relationship between sperm components across species. Without a full understanding of the general form of the different component relationships, particularly between head and flagellum length, we are limited in what can be usefully achieved.

While different sperm components have been reported to scale independently [7], we use the assumption of linear scaling as a base for our model and, as we explain elsewhere, reasons for departures from the model predictions may include this difference. At this stage the added complication of even finding consistent scaling relationships for component parts (see e.g., figure 1 of [7]) is difficult and starting with an assumption that they scale linearly is the best solution given the data available.

However, for our dataset a (non-phylogenetically corrected) model ($log(L_f) = c_1 log(L^{c_2})$, where c_1 and c_2 are the intercept and slope respectively) suggests a linear relationship between total and flagellum length is a valid simplification (Fig. S1). We modified our dataset as we found a number of bird studies where midpiece and flagellum length were recorded separately, but total length as estimated by $L = L_h + L_m + L_f$ exceeded the measured total length. We therefore chose to use only three morphological measures for this analysis: L, L_h and L_f and implemented the following rules: (1) measured L was treated as valid for all species; (2) L_h was estimated as measured L_h + measured L_m for non-aves; (3) measured L_h was treated as valid for all aves, while L_m was assumed to be included in L_f. Regressions of these component lengths against cell length provided the following relationships,

$$L_h = e^{0.475} \times L^{1.145} \text{ with } F_{1,139} = 171.3,\ p < 0.001,\ \text{adj. } r^2 = 0.549$$

$$L_f = e^{-0.965} \times L^{0.921} \text{ with } F_{1,139} = 1229,\ p < 0.001,\ \text{adj. } r^2 = 0.898$$

These two equations allow us to simulate the allometric relationship between sperm length components and drag of the sperm using equations S4-5. We are also able to show that for a range of assumed flagellar radii (a) that our treatment of drag as a function of L and not $L_h + L_f$ is appropriate.

For ease of plotting, we remove the effect of speed from the underlying drag equations (eqns. S4-5) to give a 'drag component' value (in Kg per s). Manually varying a from 0.1 to 30% of L_f allows us to examine the relationship between L and drag for the often unknown (in our dataset) value of a. As an example, Mendonca et al. [8] give the diameter of the flagellum in zebra finches as 0.293 μm, which is around 0.5% of flagellum length (55 μm [9]). Figure S3 illustrates the relationship between 'drag component' and total cell length (L) given in Figure S2, as well as the relative contributions of L_h and L_f.

We can see that, for a range of reasonable cell dimensions and for a given speed, total cell drag is always close to a linear function of cell length, independent of the relative contribution of the head and

flagellum. This then lets us ignore the separate slopes, simplifying the analysis and allowing us to deal with models of the form $Drag \propto Length$.

References

1. Ford, W. Glycolysis and Sperm Motility: Does a Spoonful of Sugar Help the Flagellum Go Round? *Hum Reprod Update* 2006, *12*, 269 274, doi:10.1093/humupd/dmi053.
2. Dusenbery, D.B. Fitness Landscapes for Effects of Shape on Chemotaxis and Other Behaviors of Bacteria. *J Bacteriol* 1998, *180*, 5978–5983, doi:10.1128/jb.180.22.5978-5983.1998.
3. Dusenbery, D.B. *Living at Micro Scale*; Harvard University Press; Harvard University Press, 2009; ISBN 9780674031166.
4. Zapryanov, Z.; Tabakova, S. Dynamics of Bubbles, Drops and Rigid Particles. *Fluid Mech Appl* 1999, doi:10.1007/978-94-015-9255-0.
5. Happel, J.; Brenner, H. *Low Reynolds Number Hydrodynamics*;; Kluwer, 1983;
6. Gillies, E.A.; Cannon, R.M.; Green, R.B.; Pacey, A.A. Hydrodynamic Propulsion of Human Sperm. *J Fluid Mech* 2009, *625*, 445, doi:10.1017/s0022112008005685.
7. Humphries, S.; Evans, J.P.; Simmons, L.W. Sperm Competition: Linking Form to Function. *BMC Evol Biol* 2008, *8*, 319, doi:10.1186/1471-2148-8-319.
8. Mendonca, T.; Birkhead, T.R.; Cadby, A.J.; Forstmeier, W.; Hemmings, N. A Trade-off between Thickness and Length in the Zebra Finch Sperm Mid-Piece. *Proc Royal Soc B Biological Sci* **2018**, *285*, 20180865, doi:10.1098/rspb.2018.0865.
9. Knief, U.; Forstmeier, W.; Pei, Y.; Ihle, M.; Wang, D.; Martin, K.; Opatová, P.; Albrechtová, J.; Wittig, M.; Franke, A.; et al. A Sex-Chromosome Inversion Causes Strong Overdominance for Sperm Traits That Affect Siring Success. *Nat Ecol Evol* **2017**, *1*, 1177–1184, doi:10.1038/s41559-017-0236-1.

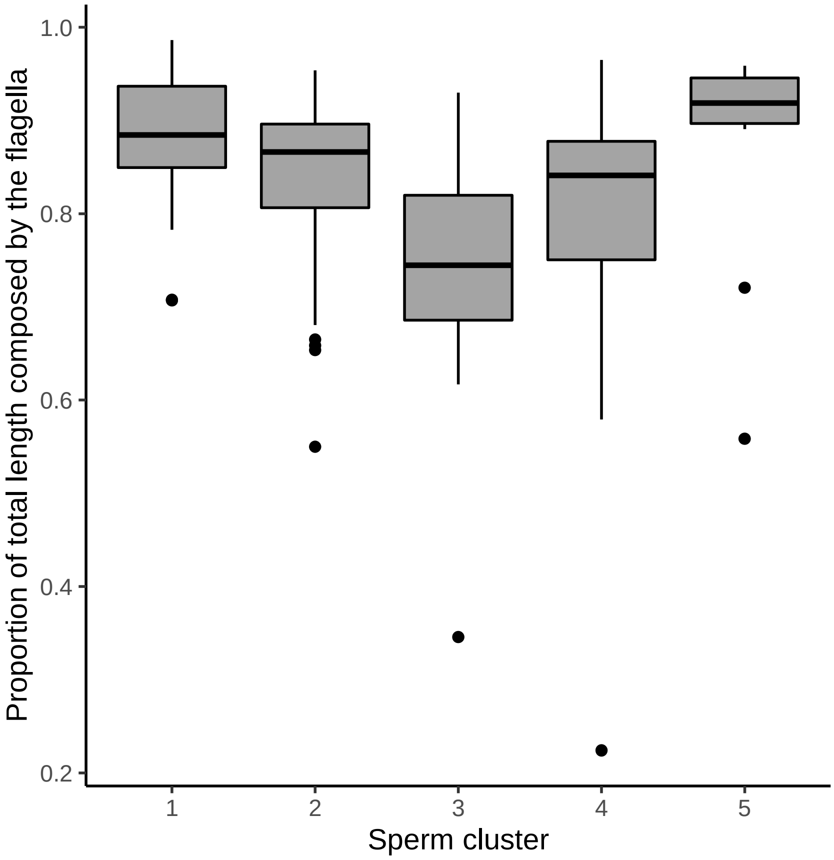

Figure S1. The relationship between proportion of total sperm length taken up by the flagella in relation to the 5 sperm clusters. In all clusters total sperm length is driven by flagella size. Outliers in the dataset may come from structurally different sperm, but also where terminology (flagella, principal piece, end piece) are misapplied.

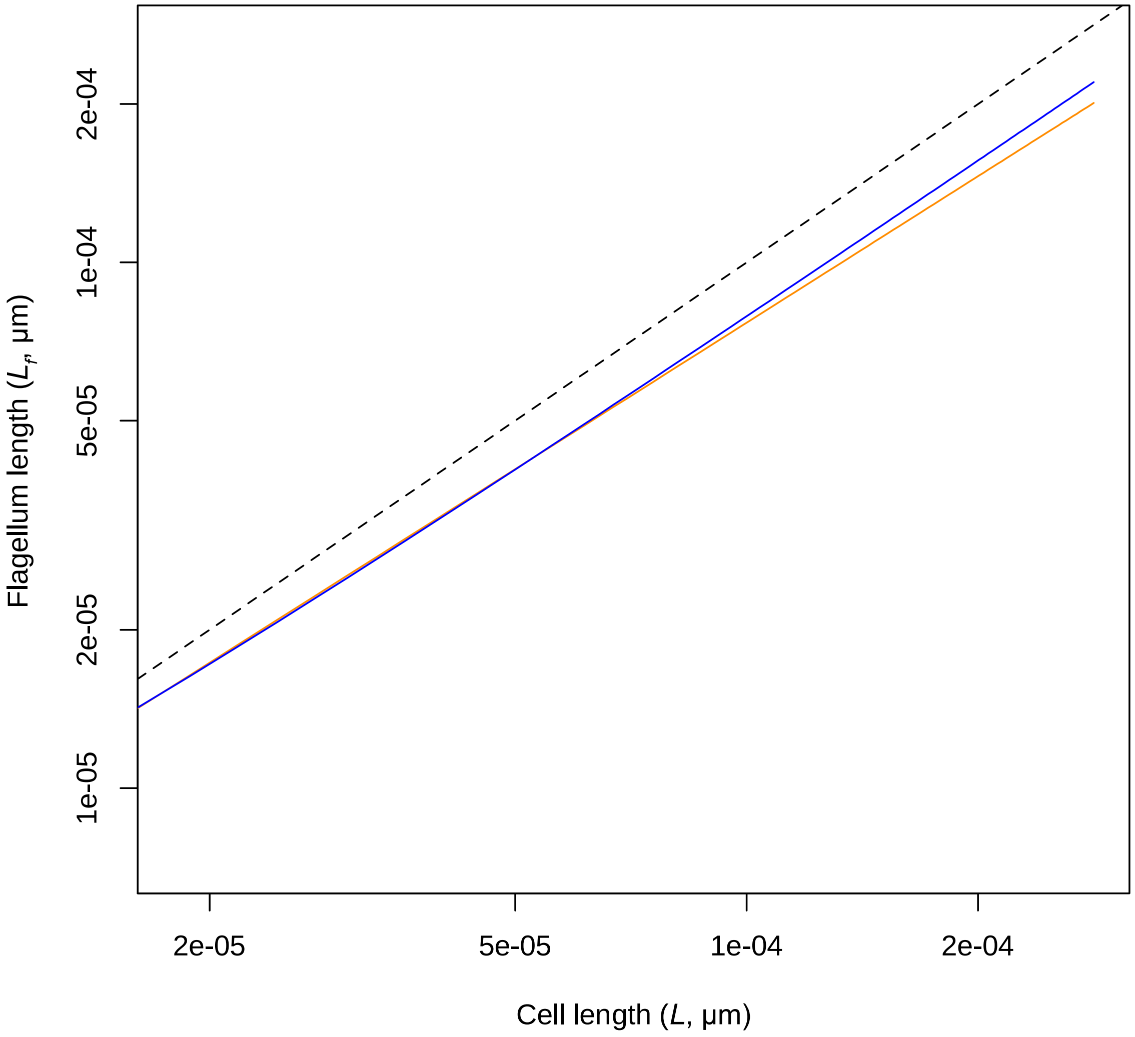

Figure S2. The relationship between total (L) and flagellar (L_f) lengths in our dataset. Dashed line is the 1:1 relationship, the data sit slightly below this line indicating that total cell length is dominated by flagellum length (i.e., $L_f >> L_h$). Coloured lines are non-phylogenetically corrected fits assuming a linear (blue) or power (orange) relationship. Linear fit, $n = 141$, $adj\ r^2 = 0.901$, $p < 0.001$; power fit ($L_f \propto L^{0.92}$), $n = 141$ $adj\ r^2 = 0.897$, $p < 0.001$.

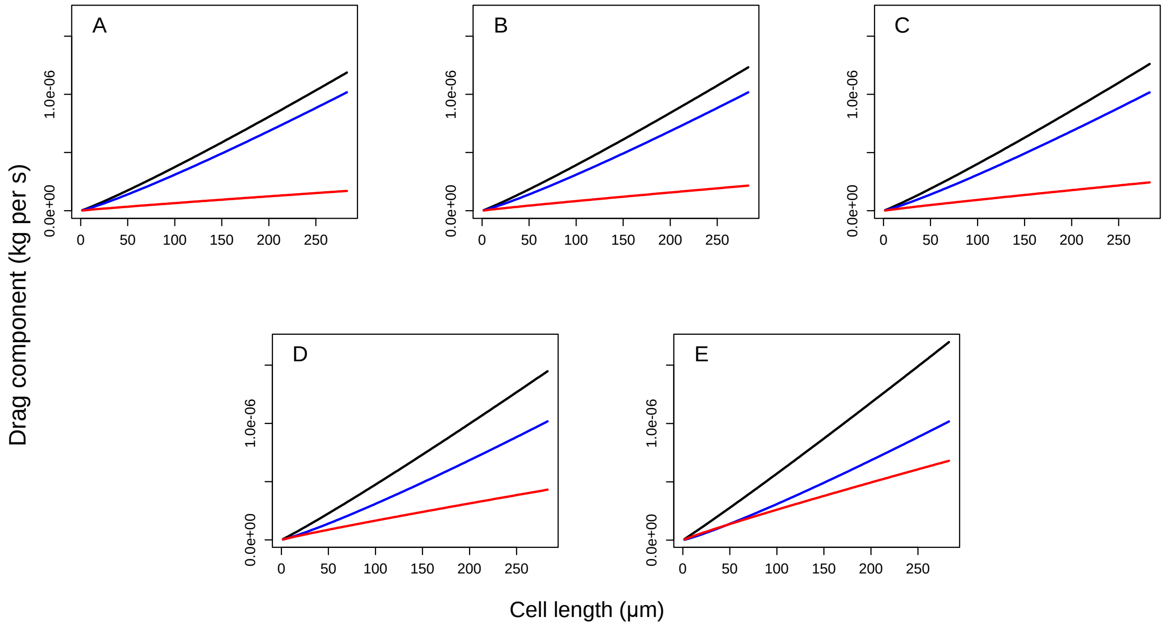

Figure S3. Simulation results for the relationship between 'drag component' and total cell length (*L*, black lines) given in Figure S2, as well as the relative contributions of L_h (blue lines) and L_f (red lines) to this drag component. Panels A-E show the effect of different ratios of flagellar radius (*a*) to *Lf*: A, *a* = 0.1% of L_f; B, *a* = 0.5%; C, *a* = 1%; D, *a* = 10%; and E, *a* = 30%. We can see that the main effect of flagellum diameter occurs as it becomes a larger proportion of the flagellum length while order of magnitude changes in the percentage when *a* is < 1% of *Lf* make little difference.

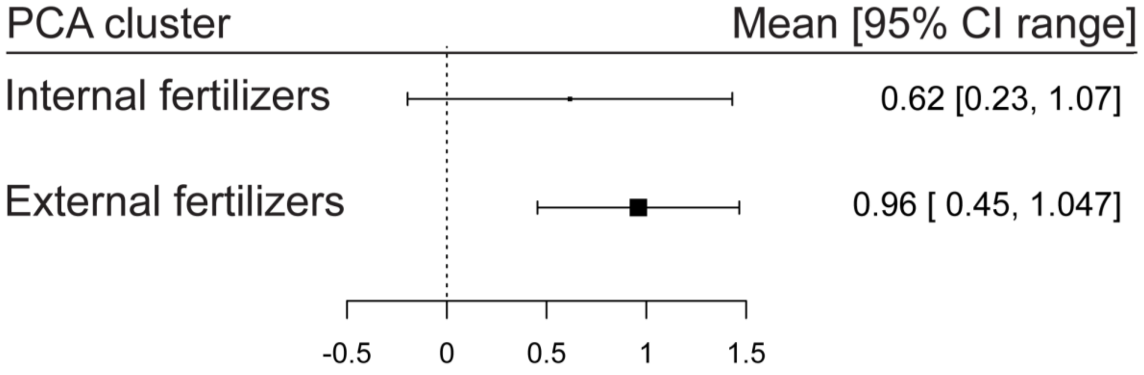

Figure S4. Speed – Flagellum length relationships for the internal and external fertilizers in PCA morphology cluster 2. Forest plot shows the distribution of mean and 95% CIs for sub-cluster slopes and point size is proportional to *n*. Only the slope for the external fertilizers differs from the expected value of zero (Constant speed model) based on 95% CI overlap**.**
